# Abnormal Voxel-Based Degree Centrality in Patients With Postpartum Depression: A Resting-State Functional Magnetic Resonance Imaging Study

**DOI:** 10.3389/fnins.2022.914894

**Published:** 2022-06-30

**Authors:** Shufen Zhang, Bo Li, Kai Liu, Xiaoming Hou, Ping Zhang

**Affiliations:** ^1^Department of Obstetrics, Shandong Second Provincial General Hospital, Jinan, China; ^2^Department of Radiology, The 960th Hospital of the PLA Joint Logistics Support Force, Jinan, China; ^3^Department of Pediatrics, Provincial Hospital Affiliated to Shandong First Medical University, Jinan, China; ^4^Department of Neurosurgery, Qi Lu Hospital, Shandong University, Jinan, China

**Keywords:** postpartum depression, voxel-based degree centrality, seed-based functional connectivity, fMRI, receiver operating characteristic (ROC) curve analysis

## Abstract

Postpartum depression (PPD) is a major public health concern with significant consequences for mothers, their children, and their families. However, less is known about its underlying neuropathological mechanisms. The voxel-based degree centrality (DC) analysis approach provides a new perspective for exploring the intrinsic dysconnectivity pattern of whole-brain functional networks of PPD. Twenty-nine patients with PPD and thirty healthy postpartum women were enrolled and received resting-state functional magnetic resonance imaging (fMRI) scans in the fourth week after delivery. DC image, clinical symptom correlation, and seed-based functional connectivity (FC) analyses were performed to reveal the abnormalities of the whole-brain functional network in PPD. Compared with healthy controls (HCs), patients with PPD exhibited significantly increased DC in the right hippocampus (HIP.R) and left inferior frontal orbital gyrus (ORBinf.L). The receiver operating characteristic (ROC) curve analysis showed that the area under the curve (AUC) of the above two brain regions is all over 0.7. In the seed-based FC analyses, the PPD showed significantly decreased FC between the HIP.R and right middle frontal gyrus (MFG.R), between the HIP.R and left median cingulate and paracingulate gyri (DCG.L), and between the ORBinf.L and the left fusiform (FFG.L) compared with HCs. The PPD showed significantly increased FC between the ORBinf.L and the right superior frontal gyrus, medial (SFGmed.R) compared with HCs. Mean FC between the HIP.R and DCG.L positively correlated with EDPS scores in the PPD group. This study provided evidence of aberrant DC and FC within brain regions in patients with PPD, which was associated with the default mode network (DMN) and limbic system (LIN). Identification of these above-altered brain areas may help physicians to better understand neural circuitry dysfunction in PPD.

## Introduction

Postpartum depression (PPD) is a common but complex condition that affects approximately 10–20% of new mothers and has detrimental effects on mothers, infants, and their families (Nguyen et al., 2019). The risk of maternal suicide, infant abuse, and infanticide are all elevated among mothers with PPD (Lee and Chung, 2007). PPD further has a long-term negative impact on the cognitive, emotional, and behavioral development of children (Halligan et al., 2007). Due to the risks posed to the mother and the infant, the mother with PPD needs early diagnosis and treatment. Understanding the changes of PPD in brain structure, function and metabolism will help us to develop early screening, diagnosis, and targeted treatment techniques.

Resting-state functional magnetic resonance imaging (rs-fMRI) has been used to detect spontaneous neural brain activity in PPD using the amplitude of low-frequency fluctuation (ALFF) analysis (Deligiannidis et al., 2013, 2019; Chase et al., 2014) or dynamic ALFF (Cheng et al., 2022b), regional homogeneity (ReHo) analysis (Xiao-juan et al., 2011), voxel-mirrored homotopic connectivity (Zhang et al., 2020), dynamic or static functional connectivity (FC) (Cheng et al., 2022b), functional connectivity density (FCD) (Cheng et al., 2021), and functional connectivity strength (FCS) (Cheng et al., 2022a). Compared with healthy controls (HCs), mothers with PPD showed significantly increased ReHo in the posterior cingulate and medial frontal gyrus and decreased ReHo in the temporal gyrus (Xiao-juan et al., 2011). The depressed mothers also showed reduced connectivity among the anterior cingulate cortex (ACC), amygdala, hippocampus, and dorsolateral prefrontal cortex, between the corticocortical and corticolimbic regions (Deligiannidis et al., 2013), between the posterior cingulate cortex (PCC) and amygdala (Chase et al., 2014), and between the dorsomedial prefrontal cortex (dmPFC) and the precuneus, posterior cingulate cortex, and supramarginal gyrus/angular gyrus regions (Deligiannidis et al., 2019). However, they showed increased connectivity between dmPFC and the rest of the default mode network (DMN) (Deligiannidis et al., 2019). Decreased voxel-mirrored homotopic connectivity values in the bilateral dmPFC, dorsal anterior cingulate cortex (dACC), and orbitofrontal cortex were observed in patients with PPD (Zhang et al., 2020). Mothers with PPD exhibited increased static FC (sFC) between the subgenual anterior cingulate cortex (sgACC) and ventral anterior insula and disrupted sFC between the sgACC and middle temporal gyrus. The changes in dynamic FC between the sgACC and superior temporal gyrus could differentiate PPD and HCs (Cheng et al., 2022b). Patients with PPD showed specifically weaker long-range FCD in the right lingual gyrus (LG.R), functional couplings between LG.R and dmPFC, and left precentral gyrus, and specifically stronger functional coupling between LG.R and right angular. Moreover, the altered FCD and resting-state FC were closely associated with depression and anxiety symptoms load (Cheng et al., 2021). The PPD group showed specifically higher FCS in right parahippocampus, and perceived social support mediated the influence of FCS in the right cerebellum posterior lobe on depression and anxiety symptoms (Cheng et al., 2022a). These studies can help clarify how PPD may affect a mother’s baseline brain activity at rest and provide a more comprehensive understanding of neural circuitry dysfunction in mothers with PPD.

The above studies focus on regional functional connectivity or analyze neural networks between selected brain regions based on a prior assumption (Deligiannidis et al., 2013, 2019). To better understand the changes in neural circuitry in PPD, we employed degree centrality (DC) to measure the global connectivity at the voxel level. DC is a new emerging reliable and compelling graph-based analysis method (Xia and He, 2017), which can identify that the voxels showed altered direct connections to all other voxels with high sensitivity, specificity, and reproducibility. It does not depend on the selection of brain regions based on prior assumptions (Bullmore and Sporns, 2009). Degree centrality (DC) has been applied to brain network research, and its abnormalities have been found in various mental disorders, such as schizophrenia (Li X. et al., 2019), major depressive disorder (Sheng et al., 2018), bipolar disorder (Deng et al., 2019), multiple sclerosis (Eijlers et al., 2017), Alzheimer’s disease (AD) (Guo et al., 2016), epilepsy (Ren et al., 2019), and Parkinson’s disease (Li M. et al., 2019). However, the DC analysis cannot provide detailed information regarding the connectivity between a voxel and the particular regions that were changed. In this study, we further conducted a seed-based FC analysis using the regions with high DC values as seeds to comprehensively explore the intrinsic abnormal connectivity of the whole-brain functional network. We tested the following hypotheses: (1) the PPD group showed abnormal DC in several brain regions compared with HCs; (2) the alterations of DC would be related to clinical symptoms; and (3) the brain regions with abnormal DC showed the aberrant FC with other brain regions.

## Materials and Methods

### Participants

The ethics committee of the Shandong Second Provincial General Hospital approved this study, and all participants provided written informed consent. Twenty-nine right-handed patients with PPD were recruited from the Department of Obstetrics of Shandong Second Provincial General Hospital and the Department of Obstetrics of the 960th Hospital of the PLA Joint Logistics Support Force. Two experienced senior associate chief physicians of neurology confirmed their diagnoses using the Structured Clinical Interview for Diagnostic and Statistical Manual of Mental Disorders, Fifth Edition (DSM-V) and Chinese Classification and Diagnostic Criteria of Mental Disorders, 3rd edition (CCMD-3). Inclusion criteria for patients were as follows: (a) their age ranged from 21 to 38 years, in the fourth week after delivery; (b) they were current first-episode, treatment-naive patients with PPD; (c) they had an Edinburgh postpartum depression scale (EPDS) score ≧12; (d) they had no other medical or mental illness history, (e) they were not substance abusers or substance dependent; (f) there were no contraindications of an MR examination; and (g) there were no organic abnormalities for MRI routine series. The EPDS scale was assessed in 1 h before the image acquisition.

A total of thirty right-handed, age-matched healthy postpartum women were recruited from the department of obstetrics. Inclusion criteria for the healthy postpartum group were as follows: (a) they were aged from 21 to 38 years and in the fourth week after delivery; (b) they did not have a current or previous history of depressive episodes; (c) their EDPS score was<3; (d) they had no other medical or mental illness history; (e) there were no substance abusers or substance dependent; (f) there were no contraindications of the MR examination; and (g) there were no organic abnormalities for the MRI routine series.

### Image Acquisition

All brain imaging data were acquired on a 3.0 T MR system (Discovery MR750, General Electric, Milwaukee, WI, United States) with a standard eight-channel head coil. During scanning, all subjects were instructed to lie still and awake, close their eyes, and breathe steadily. Special nonmagnetic foam pads were used to fix the head and minimize head movement.

High-resolution structural T1-weighted scan (Three-dimensional Brain Volume, 3D BRAVO) was performed with the following parameters: time repetition (TR) = 8.2 ms, time echo (TE) = 3.2 ms, flip angle = 12°, field of view (FOV) = 240 mm × 240 mm, slices = 115, voxel size = 1 mm, and thickness = 1.0 mm. Resting-state BOLD MR images were acquired with the following parameters: TR = 2,000 ms, TE = 30 ms, flip angle = 90°, FOV = 240 mm × 240 mm, resolution = 64 × 64, thickness = 4.0 mm, no interspace, slices = 41, gradient echo-planar volumes = 200, and duration was 6 min 40 s. In addition, T1 and T2-weighted images were collected to exclude anatomic abnormality and brain diseases for each subject.

### Functional Image Preprocessing

The fMRI data preprocessing was conducted using the Data Processing Assistant for Resting-State fMRI (DPARSF) and RESTing-state fMRI data analysis toolkit (REST)^1^, which is based on Statistical Parametric Mapping (SPM12).^2^ First, the first 10 time points of resting-state image data were discarded to ensure steady-state longitudinal magnetization. Second, the slice-time corrected images were realigned to the first volume for head motion correction. Then, T1 images were coregistered to the realigned functional images and segmented to gray matter, white matter, and cerebrospinal fluid. We normalized the resulting images to a standard Montreal Neurological Institute (MNI) template in the Montreal Neurological Institute space by applying the parameters of structural image normalization and resampling the normalized images to 3 mm isotropic voxels. After linear trend removal, the data were band-pass filtered (0.01–0.08 Hz) to eliminate physiological noise. Several sources of spurious covariates along with their temporal derivatives, including the six head motion parameters, global mean, white matter, and cerebrospinal fluid, were removed. Then, the time series of each subject was used to compute the DC.

### Degree Centrality Calculation

We computed voxel-wise DC using Pearson correlations with the REST 1.8 toolbox. The time course of each voxel in the gray matter (GM) mask was extracted and correlated with every other voxel within the mask to generate a correlation matrix (Supplementary Figure 1 and Table 1). The threshold for the Pearson’s correlation coefficient was set at *r* > 0.25 (Supplementary Figure 2). DC was computed as the sum of the weights of connections (weighted) for each voxel (Supplementary Figure 3). The resulting DC maps were spatially smoothed with a 4 mm × 4 mm × 4 mm FWHM Gaussian kernel and were improved in normality using the Fisher-z transformation. To validate the main results that did not depend on the selection of correlation thresholds, we also computed the DC maps using other different correlation thresholds (i.e., 0.1, 0.2, 0.3, and 0.4) and then reperformed statistical analysis. We found that the choice of these thresholds did not have a significant impact on the main results.

**TABLE 1:** Demographic and clinical characteristics of participants.

	Healthy control (HC, *n* = 30)		Postpartum depressed (PPD, *n* = 29)		
Characteristic	Mean (SD)	Percent (%)	Mean (SD)	Percent (%)	*P*-value
Age (years)	27.33 (4.10)		27.24 (3.55)		0.99^a^
Primipara	26	86.66	25	86.21	0.96^b^
Caesarean	9	30.0	11	37.93	0.52^b^
Breastfeeding	30	100	29	100	
**Socioeconomic status**					
(Thousand RMB)	133.0 (2.47)		144.48 (2.20)		0.06^a^
Education (years)	12.23 (2.58)		13.00 (2.15)		0.68^a^
**Neuropsychological tests**					
EPDS	0.50 (0.73)		15.79 (1.86)		0.00^a^
PSQI	6.52 (3.02)		15.17 (2.96)		0.00^a^

*SD, standard deviation; RMB, Renminbi; EPDS, Edinburgh postpartum depression scale; PSQI, Pittsburgh sleep quality index.*

*^a^Unpaired t-test, bχ2.*

### Functional Connectivity Analysis

The whole-brain cluster with significant abnormal DC in patients with PDD (compared with control subjects) was selected as seeds. We obtained FC maps by calculating the correlation coefficient (*r* score) between the mean time series of each seed region and the rest of the brain. Finally, FC maps were converted to *z*-score maps using Fisher’s *z* transformation to improve the normality. Correction for between-group FC comparisons was conducted using REST1.8 software *via* the Gaussian random field (GRF) theory correction program (voxel *p* < 0.05, cluster *p* < 0.05, 2-tailed).

### Statistical Analysis

The variables, including age and clinical symptom scores between the PPD and control group, were analyzed using the Mann–Whitney *U* test using SPSS 18.0 (SPSS Inc., Chicago, IL, United States). The differences in delivery method and time were determined using chi-square tests. The threshold was set at *p* < 0.05 (two-tailed). With age as covariates, two sample *t*-tests were performed in REST1.8 software to determine significant voxel-based differences in the DC value between the two groups. Correction for multiple comparisons was conducted using REST1.8 software *via* the GRF theory correction program within the whole brain (voxel *p* < 0.001, cluster *p* < 0.05, 2-tailed). Since DC calculation is very important in this study, we increased the *p-*value (*p* < 0.001) when doing GRF correction.

In addition, we performed Pearson correlation analyses between the DC and neuropsychological test scores of patients with PPD. We used the receiver operating characteristic (ROC) curve analysis of DC values of brain regions showing differences between the two groups to determine the brain regions’ diagnostic significance for PPD. The threshold was set at *p* < 0.05. The peak voxel coordinates with the highest significance within the brain areas of altered FC were described in terms of standard Montreal Neurological Institute coordinates. The software ‘‘BrainNet Viewer’’ in REST^3^ was used to draw a 3D brain figure.

## Results

### Demographic and Clinical Characteristics

The demographic and clinical characteristics of all subjects are listed in Table 1. There were no significant differences in age, delivery time, delivery method, feed options, socioeconomic status, or education level between PPDs and controls (*p* > 0.05). PPD groups had higher EPDS and PSQI scores (*p* < 0.001) than the HCs.

### Degree Centrality Analysis

**FIGURE 1:**
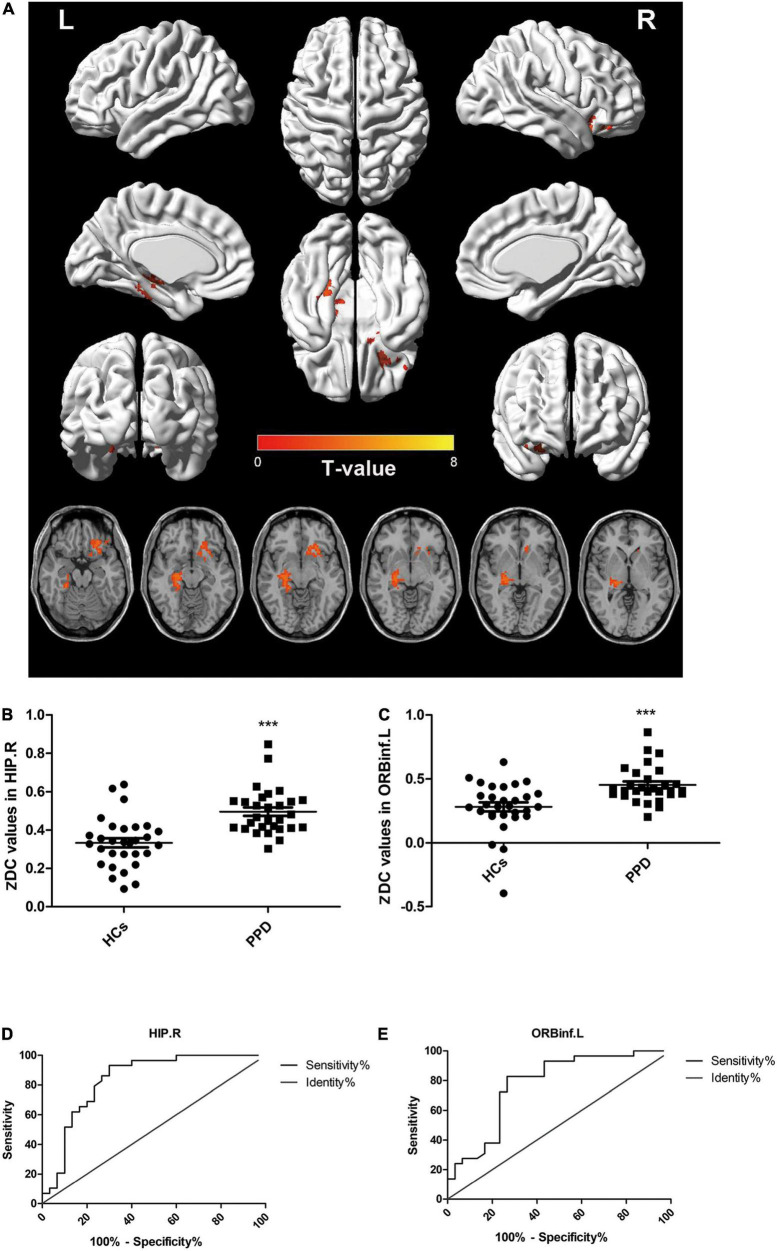
Comparisons of degree centrality between patients with postpartum depression (PPD) and healthy controls (HCs). **(A)** Brain regions with different degree centrality (DC) values between groups: HIP.R and ORBinf.L. **(B,C)** The distribution and comparison of DC values of brain regions in the PPD and HCs. **(D,E)** The ROC curve evaluates the diagnostic value of the DC value of different brain regions to distinguish patients with PPD from healthy mothers. HIP.R, right hippocampus; ORBinf.L, left inferior frontal orbital gyrus.

Compared with the HCs, the PPD group showed increased DC in the right hippocampus (HIP.R) and left inferior frontal orbital gyrus (ORBinf.L) (Figures 1A–C and Table 2). The brain areas with decreased DC were not found in PPDs compared with the HCs.

**TABLE 2:** Brain regions showing significant differences in the degree centrality between postpartum depression (PPD) and healthy controls (HCs).

Brain region	Peak MNI coordinates			Cluster size	Peak *T* value
	x	y	z	(mm^3^)	
Right hippocampus	27	−21	−6	208	3.74
Frontal_Inf_Orb_L	−24	27	−18	146	3.19

*MNI, Montreal Neurological Institute.*

The ROC curve analysis was used to test the diagnostic value of two brain regions (cluster1: HIP.R; cluster 2: ORBinf.L) with significantly altered DC between groups. The area under the curve (AUC) includes the HIP.R 0.8374 and ORBinf.L 0.7764 (Figures 1D,E).

There were no significant correlations between the DC values in the two brain regions (HIP.R and ORBinf.L) and any scores (EPDS and PSQI) in the PPD group.

### Seed-Based Functional Connectivity Analysis

**FIGURE 2:**
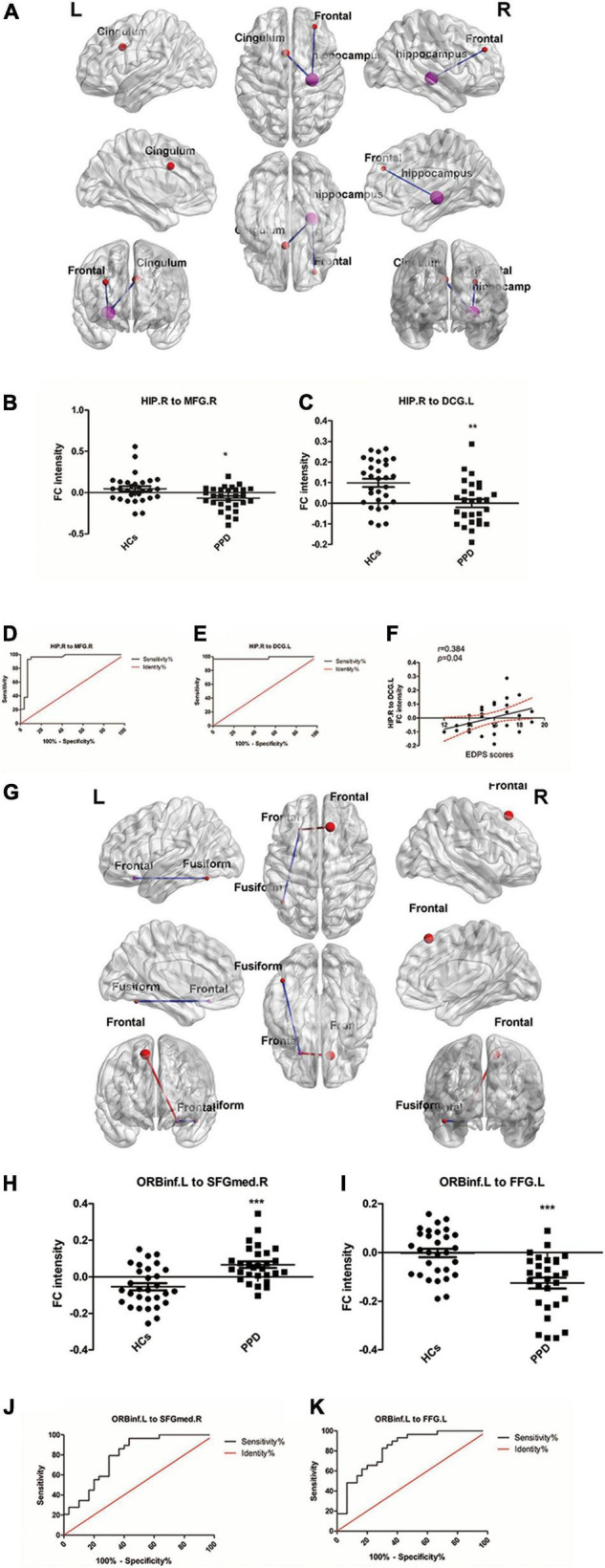
**(A)** Brain regions showing aberrant functional connectivity (FC) with HIP.R (seed region) in the PPD group compared with the HCs. Cool color represents significantly decreased FC. **(B,C)** Mean values of the abnormal functional connectivity in these groups. **(D,E)** The ROC curve evaluates the diagnostic value of the FC value of different brain regions to distinguish patients with PPD from healthy mothers, in which HIP.R was as seeds. **(F)** Scatter plots depicting a partial correlation between the HIP.R-related functional connectivity in the DCG.L and the EDPS scores for patients with PPD. **(G)** Brain regions showing aberrant FC with ORBinf.L (seed region) in the PPD group compared with the HCs. Warm color represents significantly increased FC, and cool color represents significantly decreased FC. **(H,I)** Mean values of the abnormal functional connectivity in these groups. **(J,K)** The ROC curve evaluates the diagnostic value of the FC value of different brain regions to distinguish patients with PPD from healthy mothers, in which ORBinf.L was as seeds. HIP.R, right hippocampus; ORBinf.L, left inferior frontal orbital gyrus; MFG.R, right middle frontal gyrus; DCG.L, left median cingulate and paracingulate gyri; FFG.L, left fusiform; SFGmed.R, right superior frontal gyrus, medial. ***Significant at 0.001 level and *significant at 0.05 level.

We used HIP.R and ORBinf.L as seeds in the functional connectivity analysis of the whole brain. In the PDD group, the HIP.R showed significantly decreased FC with the right middle frontal gyrus (MFG.R) and the left median cingulate and paracingulate gyri (DCG.L) compared with HCs. Furthermore, in the PPD group, the ORBinf.L showed increased FC with the right superior frontal gyrus, medial (SFGmed.R), while decreased FC with the left fusiform (FFG.L) compared with HCs (Figures 2A–C,G–I and Table 3).

**TABLE 3:** Significant differences in functional connectivity between postpartum depression (PPD) and healthy controls (HCs).

Seed area	Area with altered FC	Peak MNI coordinates			Cluster size	Peak *T* value
		x	y	z	(mm^3^)	
Right	Frontal_Mid_R	30	45	30	91	−2.98
hippocampus	Cingulum_Mid_L	−6	12	33	148	−3.17
Frontal_	Fusiform_L	−45	−60	−18	172	−3.44
Inf_Orb_L	Frontal_Sup_Medial_R	12	30	57	590	3.46

*MNI, Montreal Neurological Institute.*

The ROC curve analysis was used to test the diagnostic value of the four significant different FCs (HIP.R to MFG.R; HIP.R to DCG.L; ORBinf.L to SFGMED.R; and ORBinf.L to FFG.L) between groups. The area under the curve (AUC) includes the HIP.R to MFG.R: 0.9397; HIP.R to DCG.L: 0.9816; ORBinf.L to SFGMED.R:0.7920; and ORBinf.L to FFG.L:0.8241 (Figures 2D,E,J,K).

Correlation analysis revealed that FC intensity between HIP.R and the DCG.L positively correlated with the score of EDPS in patients with PPD (*r* = 0.384, *p* = 0.04; Figure 2F). There were no significant correlations among the FC intensity among any other regions and any other scores (EPDS and PSQI) in the PPD group.

## Discussion

This study observed voxel-level whole-brain FC abnormalities in patients with PPD using both DC and seed-based FC approaches. In this study, we found the following: (1) compared with the HCs, the PDD group showed increased DC in HIP.R and the ORBinf.L; the ROC curve analysis showed that the AUCs of the above two brain regions are all over 0.7. (2) In the seed-based FC analyses, the PPD showed significantly decreased FC between the HIP.R and MFG.R, between the HIP.R and DCG.L, and between the ORBinf.L and FFG.L compared with HCs. The PPD showed significantly increased FC between the ORBinf.L and SFGmed.R compared with HCs. (3) In particular, the HIP.R-related FC abnormalities in the DCG.L of patients with PPD were associated with EDPS scores.

The hippocampus is the core region in the limbic system (LIN) and plays a very important role in memory and cognitive function as well as the regulation of motivation, stress, and emotion (Eichenbaum, 2013). The hippocampus is highly sensitive to stress (Thomas et al., 2007). Both normal sadness and depressive illness were reported to be linked to increases in limbic areas including the hippocampus (Fitzgerald et al., 2008; Delaveau et al., 2011). It has been reported that MDD leads to an increased nodal centrality (both degree and strength) for the right hippocampus (Chu et al., 2018); patients with MDD have impaired functional connections of the hippocampus (Gray et al., 2020). In this study, we found that higher DC in the right hippocampus in PPD, which means that the right hippocampus had the increased centrality in PPD’s brain network. However, the seed-based FC analysis showed that the right hippocampus presented weaker connectivity with the MFG.R and the DCG.L compared with HCs. It had already been observed the attenuation of connectivity between the dlPFC and hippocampus in PPD subjects (Deligiannidis et al., 2013). The results suggested that a higher DC value is not necessarily better; too high may indicate wrong connectivity or invalid connectivity. The appearance of invalid connectivity or wrong connectivity will lead to a decrease in brain function. The abnormal DC and FC of the hippocampus might explain memory deficits and the depression experienced by patients with PDD.

The MFG plays an essential role in a variety of cognitive functions, such as working memory, motor control, and attentional reorientation (Japee et al., 2015). Decreased structural and FC of MFG have been frequently reported in depressed individuals (Korgaonkar et al., 2014; Sheng et al., 2018). DCG.L is the part of the cingulate gyrus and is involved in behavior, motor, and somatosensory function, especially in emotion, information transmission, and cognitive processing (Oane et al., 2020). Aberrant activity of this brain region is associated with negative emotions (Jiang et al., 2020), episodic memory, and rumination processing of depressive symptoms (Huang et al., 2021). We found that FC intensity between HIP.R and DCG.L positively correlated with the score of EDPS in patients with PPD. We used the original FC value when doing the correlation analysis. There were 15 negative values of FC between HIP.R and the DCG.L. The negative values mean that the stronger the FC, the smaller the value, so it was positively correlated with the score. This result highlighted the importance of HIP.R and DCG.L in PPD, and the abnormal FC between them might be a distinct feature in the neurobiology of PPD. Integrative dysfunctions of these regions may contribute to disturbances in mood, cognition, and memory in PPD.

The ORBinf.L refers to one of the three parts of the inferior frontal gyrus that plays an important role in the regulation of emotion and attention (Cha et al., 2016). It is involved in behaviors related to emotion and empathy and shows increased functional activity when individuals experience subjective feelings of guilt (Briggs et al., 2019). In disease, the orbital part of the inferior frontal gyrus exhibits abnormal functional connectivity in patients with depression (Rolls et al., 2020) and anxiety (Cha et al., 2016). In this study, we found that the PPD group showed increased DC in ORBinf.L that showed increased FC with the SFGmed.R, while decreased FC with the FFG.L compared with HCs. The medial superior frontal gyrus, as an important part of the superior prefrontal gyrus, is associated with self-consciousness, self-referential processing, emotion regulation, and cognitive processing (Yan et al., 2021). It played a partial mediating role in the relationship between perceived stress and depression (Wang et al., 2019). The fusiform gyrus is involved in many aspects of cognition, especially emotion recognition in social-cognitive processes (Jung et al., 2021). The abnormal neural activity in the fusiform gyrus may be associated with the severity of depression or susceptibility to depression (Huang et al., 2021). The abnormal FC among these above regions might explain depression, anxiety, stress, and social impairments among patients with PPD.

In this study, we demonstrated that PPD-related integrative disturbances were most commonly located in the DMN and LIN. The HIP.R, MFG.R, DCG.L, ORBinf.L, and SFGmed.R were suggested as key nodes of DMN. DMN is engaged in a diverse array of functions, such as episodic memory, self-referential activity, and monitoring the self and surrounding environment (Raichle, 2015). LIN is mainly involved in memory, regulation of negative cognition, and emotion (Rolls, 2015). DMN and LIN exhibited abnormal neuro-activity and were involved in the physiopathology of depression (Sheng et al., 2018). Our results supported the preferential involvement of hubs and the DMN/LIN in PPD and developed models of network alterations in the disease, which might help better understand the underlying neurobiology of PPD. The ROC curve analysis showed that the AUC of the HIP.R and the ORBinf.L and their altered FCs were all over 0.7. The range of AUC between 0.7 and 0.9 indicates the ideal diagnostic value. The brain regions with high DC values and the abnormal FCs in PPD had appropriate diagnosis accuracy and could be used as the imaging biomarkers of patients with PPD for diagnosis.

However, this study has several limitations. First, the sample size was relatively small, which may affect statistical power. Second, DC can only identify brain regions with abnormal functional connectivity and is unable to provide a clear causal relationship. Third, this study lacks the comparison between the pretreatment and posttreatment of patients with PPD and could not provide the imaging change of the above brain areas after treatment. Fourth, it is controversial about the time of onset of PPD. We chose the fourth week, the time of the new mother’s first postpartum follow-up in the hospital, to do the EPDS scale and acquire the fMRI images. We will follow up with the mothers and do the EPDS scale and acquire the fMRI images within the first 6 weeks and 1 year after delivery in our following research to further verify our results. Fifth, the cognitive functions of the new mothers were not assessed in detail. In our following research, we will use Beck’s Anxiety Inventory (BAI), Pittsburgh Sleep Quality Index (PSQI), and SymptomChecklist90 (SCL-90) to assess the new mothers thoroughly. There is still no complete consensus on the orders between the temporal filtering and the nuisance regression during data preprocessing. In this study, linear regression was conducted after band-pass filtering the data (0.01–0.08 Hz) according to the processing procedure of similar studies (Zhang et al., 2020; Li et al., 2021; Wang et al., 2021) and the default order DPARSF and REST software. We will explore two data processing pipelines for PPD disease in future studies. In conclusion, we found abnormal DC values and FCs in a variety of brain regions in the PPD groups, which might demonstrate the reorganization of the brain network in PPD and provide imaging biomarkers for early screening and accurate diagnosis of PPD.

## Data Availability Statement

The original contributions presented in this study are included in the article/Supplementary Material, further inquiries can be directed to the corresponding authors.

## Ethics Statement

The studies involving human participants were reviewed and approved by the Shandong Second Provincial General Hospital. The patients/participants provided their written informed consent to participate in this study.

## Author Contributions

PZ and XH: conception, study design, interpretation of the results, drafting the manuscript work, or revising it critically for important intellectual content. SZ, BL, and KL: data collection or acquisition. SZ and BL: statistical analysis. All authors approved the final version to be published and agreement to be accountable for the integrity and accuracy of all aspects of the work.

## Conflict of Interest

The authors declare that the research was conducted in the absence of any commercial or financial relationships that could be construed as a potential conflict of interest.

## Publisher’s Note

All claims expressed in this article are solely those of the authors and do not necessarily represent those of their affiliated organizations, or those of the publisher, the editors and the reviewers. Any product that may be evaluated in this article, or claim that may be made by its manufacturer, is not guaranteed or endorsed by the publisher.

## Footnotes

1
http://www.restfmri.net

2
http://www.fil.ion.ucl.ac.uk/spm

3
http://www.nitrc.org/projects/bnv/
