# Supplementary material for: Abnormal Voxel-Based Degree Centrality in Patients With Postpartum Depression: A Resting-State Functional Magnetic Resonance Imaging Study
Source: Front Neurosci. 2022 Jun 30;16:914894. doi: 10.3389/fnins.2022.914894 (PMC9280356; doi:10.3389/fnins.2022.914894)
Supplement: Supplementary file 1 [file Data_Sheet_1.docx]

1. The original group-level degree centrality results for the healthy controls (Supplementary figure 1A and Supplementary table 1) and patients (Supplementary figure 1B and Supplementary table 2) showed increased DC in Postcentral_R and Precentral_L.

A

B


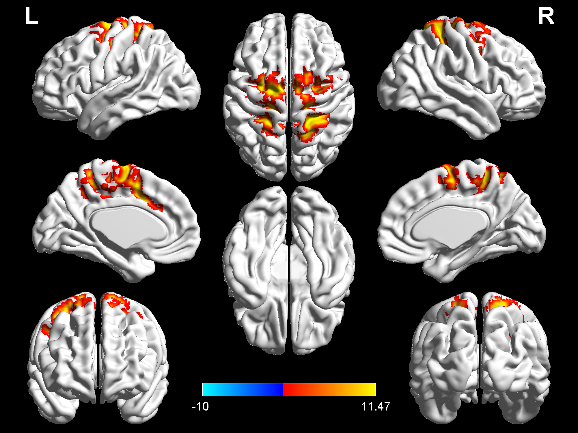


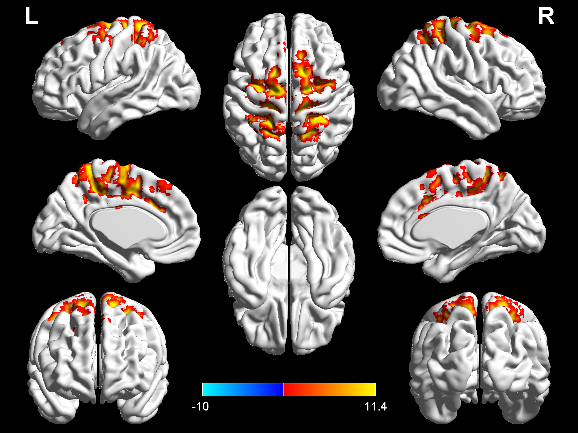


Supplementary figure 1: The original group-level degree centrality maps for the healthy controls(A) and patients(B).(A)The healthy mothers showed high DC in Postcentral_R and Precentral_L. (B)The PPD group showed high DC in Postcentral_R and Precentral_L.

Supplementary table 1 The original group-level degree centrality results for the healthy controls


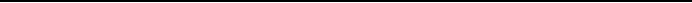


Brain Peak MNI coordinates Cluster size Peak intensity

region x y z （mm^3^）

Postcentral_R 24 -48 63 1188 11.34

Precentral_L -21 -24 69 1323 11.20

Abbreviation: MNI, Montreal Neurological Institute

Supplementary table 2 The original group-level degree centrality results for the PPD patients


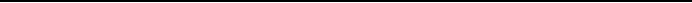


Brain Peak MNI coordinates Cluster size Peak intensity

region x y z （mm^3^）

Postcentral_R 12 -33 60 820 11.36

Precentral_L -18 -18 72 945 11.47

Abbreviation: MNI, Montreal Neurological Institute

1. To validate the main results did not depend on the selection of correlation thresholds, we also computed the DC maps using other different correlation thresholds (i.e., 0.1, 0.2, 0.3 and 0.4) and then reperformed statistical analysis. We found that the choice of these thresholds did not have a significant impact on the main results (Supplementary figure 2).


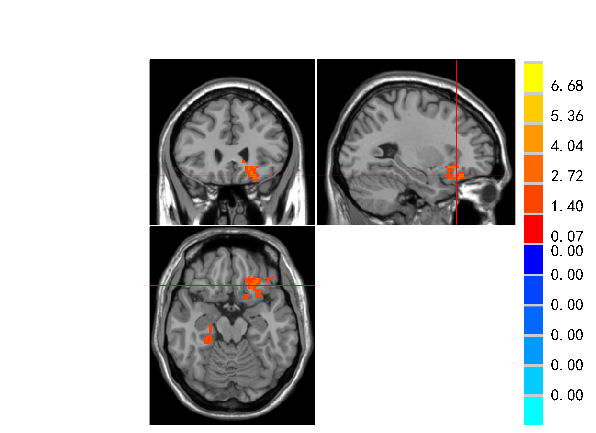

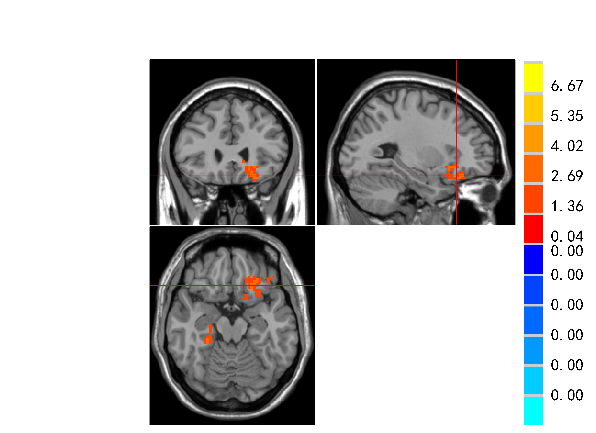


B

A


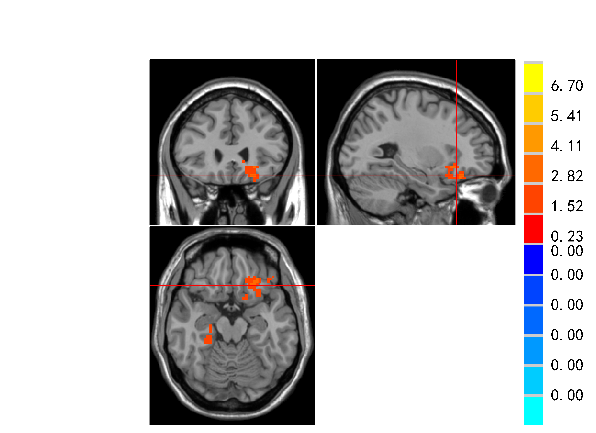

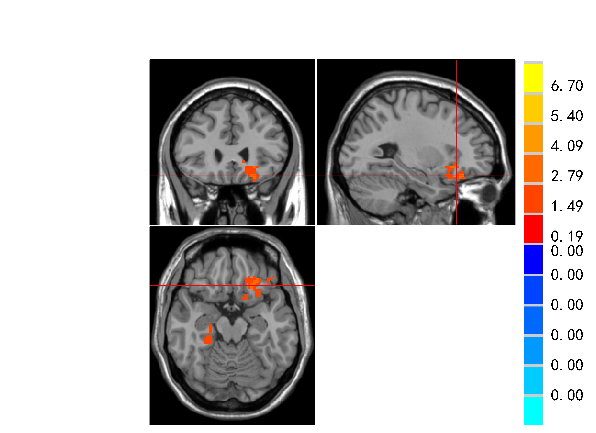


D

C


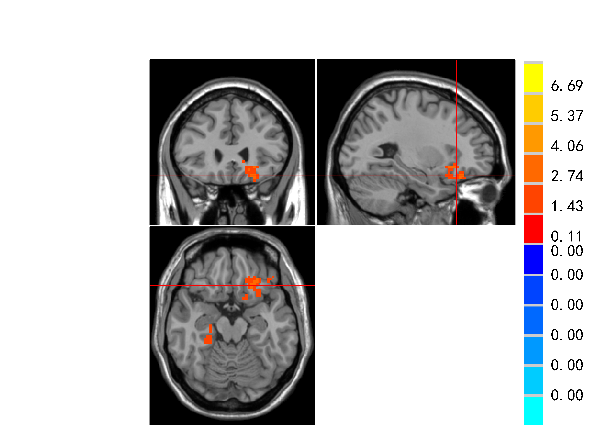


E

Supplementary figure 2: The DC results with different threshold values showed similarly. (A: 0.1, B: 0.2, C: 0.3, D: 0.4, and E: 0.25).

1. The REST software provides two calculation modes of the output of DC results. One is connections (binarized), the other is the sum of the weights of connections (weighted). The two outputs of our DC results were almost the same (Supplementary figure 3).


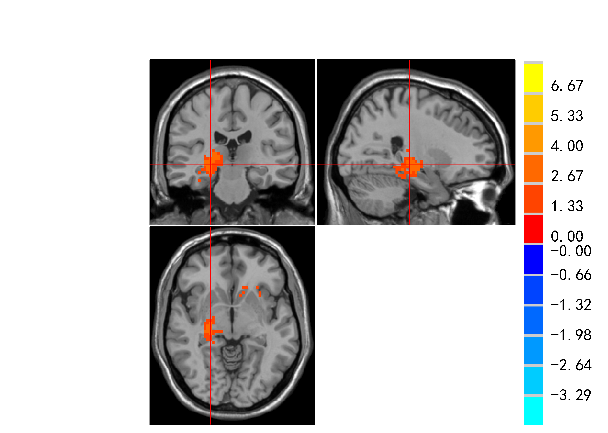

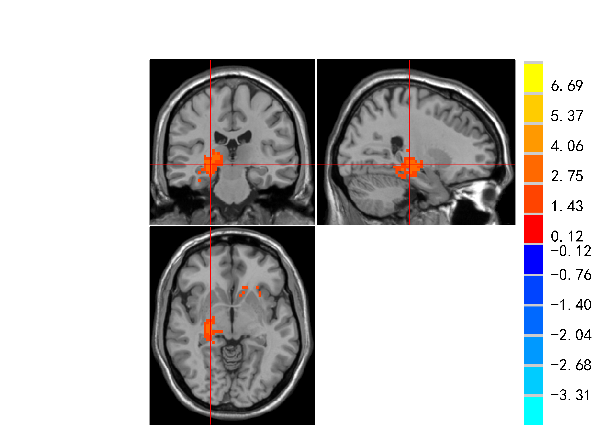


A

B

Supplementary figure 3 The DC results showed as (A) the sum of connections (binarized) or (B) the sum of the weights of connections (weighted) for each voxel.
